# Systematic Review and Meta-Analysis of Randomized Clinical Trials of Anti-Inflammatory Agents in Early-Stage Psychotic Disorders

**DOI:** 10.1093/schbul/sbaf173

**Published:** 2025-10-28

**Authors:** Edward R Palmer, Matthew J Taylor, James E Hotham, David Salam, Ghayath Alshawaf, Jack C Rogers, Rachel Upthegrove

**Affiliations:** Institute for Mental Health, School of Psychology, University of Birmingham, Birmingham, B15 2TT, United Kingdom; Early Intervention Service, Birmingham and Solihull Mental Health NHS Foundation Trust, Birmingham, B1 3RB, United Kingdom; Mental Health Research Unit, Sheffield Centre for Health and Related Research, The University of Sheffield, Sheffield, S1 4DA, United Kingdom; Sheffield Health and Social Care NHS Foundation Trust, Sheffield, S4 7QQ, United Kingdom; Institute for Mental Health, School of Psychology, University of Birmingham, Birmingham, B15 2TT, United Kingdom; Herefordshire and Worcestershire Health and Care NHS Trust, Worcester, WR5 1JR, United Kingdom; Institute for Mental Health, School of Psychology, University of Birmingham, Birmingham, B15 2TT, United Kingdom; Birmingham and Solihull Mental Health NHS Trust, Birmingham, B1 3RB, United Kingdom; Institute for Mental Health, School of Psychology, University of Birmingham, Birmingham, B15 2TT, United Kingdom; Early Intervention Service, Birmingham and Solihull Mental Health NHS Foundation Trust, Birmingham, B1 3RB, United Kingdom; Institute for Mental Health, School of Psychology, University of Birmingham, Birmingham, B15 2TT, United Kingdom; Institute for Mental Health, School of Psychology, University of Birmingham, Birmingham, B15 2TT, United Kingdom; Early Intervention Service, Birmingham and Solihull Mental Health NHS Foundation Trust, Birmingham, B1 3RB, United Kingdom; Department of Psychiatry, Warneford Hospital, University of Oxford, Oxford, OX3 7JX, United Kingdom

**Keywords:** anti-inflammatory treatment, early psychosis, first-episode psychosis, clinical high risk

## Abstract

**Background and Hypothesis:**

Accumulating evidence suggests that immune dysregulation is present in psychosis, however, evidence for anti-inflammatory treatments is mixed. This may be because studies need to focus on when inflammation offers a modifiable target. This review and meta-analysis sought to clarify the effects of anti-inflammatory agents from high-quality randomized trials in patients at clinical high risk for psychosis (CHR) and first-episode of psychosis (FEP).

**Study Design:**

Databases were searched until January 2025 for double-blind, randomized, placebo-controlled trials evaluating the effect of anti-inflammatory treatment compared with placebo in CHR and FEP populations. Primary outcomes were transition rates to psychosis in CHR and changes in total psychotic symptoms in FEP. Secondary outcomes included changes in symptoms in CHR and changes in symptom sub-scores in FEP.

**Study Results:**

Searches retrieved 2168 articles, with 17 meeting inclusion criteria (5 for CHR, 12 for FEP). In CHR, anti-inflammatory treatment was not associated with a significant reduction in transition to psychosis (odds ratio 0.88, 95% CI, 0.26-3.01, *P* = .80). In FEP, anti-inflammatory treatment demonstrated a significant reduction in total psychotic symptoms; (standardized mean differences = −0.38, 95% CI, −0.76 to 0.00, *P* = .05). Secondary outcomes showed no change in symptoms in CHR, and significant changes in Positive and Negative Syndrome Scale positive sub-scores in FEP.

**Conclusions:**

Adjuvant anti-inflammatory treatment may be efficacious in FEP. However, high heterogeneity was present across studies, with possible publication bias and small-study effects. We highlight the need for further, large, stage-specific trials to conclusively understand the potential therapeutic benefit of anti-inflammatory treatments in early psychosis.

## Introduction

Psychotic disorders, such as schizophrenia, affect approximately 1% of the population and lead to significant distress and disability.^1^ Early intervention, focusing on those at clinical high risk (CHR) for psychosis and those in their first episode of psychosis (FEP), has improved significantly over recent decades.^2^ However, limitations to treatments remain due to poor efficacy,^3^^,^^4^ tolerability^5^^,^^6^ and long-term side effects.^7^^,^^8^

As such, the search for more effective and better-tolerated medications is paramount, driven by investigation into novel treatment targets and mechanisms. One such mechanism is chronic low-grade inflammation, for which there is primary evidence of a role in psychosis. Strong meta-analytical data suggests that inflammation is present in both acute and chronic stages of psychosis,^9^ and raised inflammatory profiles have been observed specifically in CHR^10^ and FEP^11^ populations. Inflammation has also been implicated in shaping both the risk of developing psychosis and its symptom profile. For example, inflammation during childhood has been shown to be associated with an increased risk of psychosis in adulthood,^12^^,^^13^ as well as with increased negative symptoms.^11^ Other preclinical work has demonstrated mechanistic, inflammatory-mediated changes in early psychosis populations, such as heightened neuroinflammation, oxidative stress,^14^^,^^15^ and increased microglia activation.^16^^,^^17^ While further mechanistic work is needed to definitively establish causality, Mendelian Randomization studies have demonstrated a potentially causal effect of genetically predicted levels of inflammation and psychosis.^18^^,^^19^ Chronic low-level inflammation is not found in all of those with psychosis, and inflammation is hypothesized to be relevant in a subset of patients estimated to be between 30% and 50%.^20^ Together, these findings suggest that inflammation in early psychosis may represent a unique and time-sensitive target for intervention. Furthermore, studying early psychosis also reduces confounding from long-term medication use, illness chronicity, and lifestyle-related factors, offering a clearer understanding of anti-inflammatory effects.^21^

Numerous individual studies have investigated the use of anti-inflammatory medications in psychotic disorders, and these have been analyzed by several meta-analyses.^22-39^ Of the existing systematic reviews, none focus specifically on early psychosis. Two meta-analyses have examined all types of anti-inflammatory interventions across all stages of psychosis.^26^^,^^27^ The most comprehensive and recent study by Jeppesen et al.^26^ assessed primarily anti-inflammatory medications (classical anti-inflammatories) such as non-steroidal anti-inflammatory drugs (NSAIDs) or antibiotics, and medications with known or pleiotropic anti-inflammatory effects (non-classical anti-inflammatories) such as N-acetylcysteine (NAC), statins, and polyunsaturated fatty acids (PUFAs). They reported a small to medium effect size for adjunctive anti-inflammatory medication in all symptom subdomains (positive, negative, and general). However, a sensitivity analysis limited to individuals with symptom duration under 2 years did not show significant improvements. Other meta-analyses pooling across all stages of psychotic disorder have investigated the effects of single anti-inflammatories in individuals with psychosis, and have yielded conflicting results; for example, no benefit for PUFAs^24^ or NAC,^25^ and statins^22^ and positive results for estrogens,^28^^,^^31^ minocycline,^32^^,^^33^ celecoxib,^34^ and statins.^29^^,^^30^

Evidence specific to individuals with CHR is more limited. One meta-analysis, which included observational studies as well as randomized placebo-controlled trials (RCTs) reported a positive effect of PUFAs in reducing transition rates to psychosis.^23^ One major challenge faced by trials in CHR populations is that between-group differences can be difficult to detect due to low transition rates.^40^ As a result, very large sample sizes are needed to ensure sufficient statistical power.

The mixed evidence across studies and the lack of dedicated meta-analyses for early psychosis, where the signal for an inflammatory pathway may be strongest, together with a growing number of trials in this evolving field, necessitate this up-to-date focused review. We aim to comprehensively assess the evidence of the effectiveness of both classical and non-classical anti-inflammatory medications in early psychosis, specifically in CHR and FEP populations.

## Methods

The study protocol was registered with Prospero (ID 226925) prior to the initiation of the search, and amendments to the protocol are outlined in the Supplementary Section 1.

### Eligibility Criteria

Double-blind, RCTs exploring anti-inflammatory medication effects were included with no limits placed on the year or language of publication. We examined studies in 2 different populations: clinically high risk (CHR) for psychosis and first episode psychosis (FEP). No age restrictions were placed on either group.

For CHR, we included any RCT of “at-risk” participants, which included terms such as at-risk for psychosis, CHR, or ultra-high risk. To ensure standardization, CHR status had to be established using validated assessment tools, such as the Comprehensive Assessment of At-Risk Mental States (CAARMS)^41^ or Structured Interview for Psychosis-Risk Syndromes (SIPS).^42^ CHR features include, but are not limited to, genetic risk, deterioration of functioning, and higher levels of unusual thought content.^43^ For studies that potentially contained individuals with CHR, authors were contacted to determine whether data specific to the CHR subgroup could be isolated.

We defined FEP as people within the first 3 years of diagnosis of a psychotic illness as determined by individual study inclusion criteria. For studies that potentially included individuals with FEP, authors were contacted to determine whether data specific to the FEP subgroup could be isolated. If it was not possible to isolate individuals with a duration of illness under 3 years, the entire cohort was included if the study population had a maximum duration of illness less than 5 years and a mean duration of illness less than 3 years.

### Interventions

We included any medications with known anti-inflammatory effects (classical anti-inflammatories) such as NSAIDs or antibiotics, and medications with known or theorized secondary anti-inflammatory effects such as NAC, and PUFAs (non-classical anti-inflammatories). Anti-inflammatory interventions were compared with placebo and treatment as usual. Trials were eligible for inclusion irrespective of whether anti-inflammatory treatments were deployed as standalone or adjunctive therapies, for example, in combination with antipsychotic medications.

### Outcome Measures

The primary outcome for CHR was the rate of transition to psychosis, as measured by a standardized assessment (eg, CAARMS, or SIPs). The secondary outcome was the change in total psychotic symptom severity as measured in reporting studies. For FEP, the primary outcome was change in total psychotic symptom severity from baseline to the primary endpoint of each study. Symptom severity was measured using the total score of either the Positive and Negative Syndrome Scale (PANSS)^44^ or the Brief Psychiatric Rating Scale (BPRS).^45^ Secondary outcomes were subscale scores (e.g., PANSS positive, negative, and general sub-scores). We also examine the effect of different anti-inflammatory classes, both of each individual type of anti-inflammatory and classical vs non-classical anti-inflammatories. These distinctions are clinically relevant and align with previous reviews, such as Jeppesen et al.^26^ facilitating meaningful comparisons across studies.

### Search Strategy

Two authors (ERP and MJT) searched CENTRAL (Cochrane), EMBASE (Ovid), MEDLINE (Ovid), PubMed, and PsycINFO (APA) from inception to November 22, 2023, for double-blind, placebo-controlled, RCTs evaluating the effect of classical or non-classical anti-inflammatory medications in participants with early psychosis. Full search terms are listed in the Supplementary Section 2, but in brief, the search took the form of RCT AND (FEP OR CHR) AND (anti-inflammatory). Search results were manually de-duplicated using reference manager software before being imported into Covidence (Veritas Health Innovation, Melbourne, Australia). Further de-duplication occurred using automated de-duplication functionality incorporated within Covidence. A repeat search was conducted in January 2025 to identify papers published since the original search. A full search strategy can be seen in the Supplementary Section 2.

### Study Selection

In total, 5 authors contributed to study selection (ERP, MJT, JH, DS, and GA). During title and abstract screening, each study article was reviewed by 2 independent reviewers. A third reviewer was consulted for any conflicts. An identical process was followed for full-text reviews, with any ongoing queries discussed among all reviews to achieve consensus. Study authors were contacted if the duration of illness was unclear or if the sample could have potentially contained people meeting the criteria for FEP or CHR. Authors were also contacted for conference abstracts, posters, trial registrations, or protocol papers, where no apparent follow-up publication had reported final results, and to ask whether any results were available for inclusion. For further details on the requests made, see the Supplementary Section 3. One of the studies which was screened after meeting all inclusion criteria was published in Russian. The paper was translated into English using Google Translate, with the quality of this translation verified by a native Russian-speaking colleague with medical training (VD).

### Data Extraction

Data were extracted independently by 2 reviewers, and any conflicts were resolved through consensus. Where multiple reports corresponding to the same data were highlighted, the primary paper reporting relevant outcomes was used. Data were collected on all primary and secondary outcomes, where available, in addition to trial registration, funding, study aims, study setting, randomization details, blinding details, use of antipsychotic medication alongside anti-inflammatory intervention, study outcomes, inclusion and exclusion criteria, participant data such as gender, age, duration of illness, ethnicity, detail of the interventions and placebo such as dosing, regime, and length of intervention.

### Risk of Bias Assessment

Risk of bias assessment was done using Version 2 of the Cochrane Risk of Bias tool for randomized controlled trials (ROB2).^46^^,^^47^ The ROB2 assesses for risk of bias in 5 domains: randomization, deviations from intended interventions, missing outcome data, measurement of outcome, and reporting of results. Each study is scored as either low, some concerns, or high risk of bias. An overall judgment is then made based on the outcomes of the different domains. This is usually set at the level of the highest individual domain outcome (meaning that if any domain outcome is high, the overall outcome will be high). Each study was reviewed independently by 2 reviewers. Any conflicts were discussed and resolved by ERP, MJT, and RU.

### Statistical Analysis

#### Effect Measures

For binary outcomes, such as transition to psychosis in CHR, we used odds ratios (ORs) to compare rates between intervention and control arms. For continuous outcomes, such as change in mean psychotic symptom severity from baseline to the primary endpoint in FEP, we compared mean differences, with SDs, between intervention and control arms. The mean difference represents an average change in points on the respective scales. Where outcomes were measured using more than 1 symptom scale (e.g., PANSS or BPRS), scores were harmonized using standardized mean differences (SMD), calculated in Revman with bias-correction using Hedges’ g.^48^^,^^49^ Secondary outcomes, looking at changes in sub-scores, were measured as PANSS scores in all instances.

#### Handling of Missing Data

For FEP, not all studies directly reported the change in psychotic symptom severity (mean differences) with associated SDs. Seven studies^50-56^ provided only baseline and endpoint symptom scores with SDs. For these, mean differences and SDs were calculated from baseline and endpoint values.^48^ Two studies^51^^,^^57^ did not report the full set of PANSS scores; in these cases, the missing sub-scores or total scores with SDs were calculated. Where standard errors (SEs) were reported, these were converted to SDs. For further details on these processes, please refer to the Supplementary Section 4.

### Statistical Synthesis Methods

Statistical analysis was conducted using Revman (web version 8.14.0; https://revman.cochrane.org/). A random effects model was used as we predicted generally high levels of heterogeneity between the studies due to differences in cohort sizes, interventions, medications, and follow-up duration. Inverse variance weighting methods were used for all estimates. Between-study variance was estimated using the restricted maximum likelihood method,^58^^,^^59^ and heterogeneity was quantified using Tau.^2^ Summary effect CIs used Hartung-Knapp-Sidik-Jonkman (HKSJ) as the between-study estimate is greater than zero.^60^ Heterogeneity was further explored using the Metafor package (version 4.8-0) in R Studio,^61^ with meta-regression used to explore potential factors driving heterogeneity. Three studies included more than a single intervention.^52^^,^^62^^,^^63^ We followed the Cochrane handbook^64^ advice for how to address these individually, and this has been outlined in the Supplementary Section 5. These studies were excluded from sensitivity analyses looking at individual types of anti-inflammatories.

### Sensitivity Analysis

Several sensitivity analyses were conducted to assess the robustness of findings. First, the effects of study size, year of publication, duration of treatment, mean age of participants, type of anti-inflammatory, and risk of bias on heterogeneity were assessed via meta-regression analysis. The effect of risk of bias was further explored using subgroup analyses, comparing results across low, moderate, and high risk of bias categories, and excluding studies with a high ROB. Second, to assess the influence of individual studies, a leave-one-out analysis was performed, systematically removing each study and recalculating the pooled effect size. Third, small study effect and risk of publication bias were assessed using funnel plots, Egger’s test,^65^ and a trim-and-fill analysis.^66^ Fourth, to aid comparison with other reviews, a meta-analysis limited to studies with a duration of illness of less than 2 years was performed. Finally, variance was assessed by comparing standard deviations across studies and examining the coefficient of variation. Examining measures of variance can help detect heterogeneity in response, indicating whether certain subgroups may be driving the treatment effect with a high degree of variance, suggesting that participants respond differently to the intervention, with some experiencing strong effects while others show little to no response. For further details on sensitivity analysis methods, please see the Supplementary Section 7.

### Certainty of Evidence

We applied the Grading of Recommendations Assessment, Development and Evaluation (GRADE) assessment^67^ to assess the certainty of evidence of primary outcomes, following standard methodological criteria evaluating the risk of bias, inconsistency, indirectness, imprecision, and other considerations, including publication bias. Assessments were done independently by ERP and MJT, and any conflicts were resolved between them. See Supplementary Section 8 for further details.

## Results

A total of 2168 studies were retrieved during initial searches. Following manual and automated deduplication, 1363 studies underwent title and abstract screening, following which 315 underwent full-text review. Repeat searches in January 2025 identified a further 87 articles, 8 of which underwent full-text review, with 3 further studies identified as meeting all inclusion criteria. Overall, 5 CHR and 12 FEP studies met the inclusion criteria. In all FEP trials, anti-inflammatory agents were used as adjuncts to antipsychotic medication, however, in four of the CHR studies, anti-inflammatories were used in isolation (see Table 1 and the Prisma flow diagram Figure 1).

**Table 1:** Summary Table of Included Studies.

**Study (author and year)**	**Population**	**Drug**	**Daily dose**	**Antipsychotic** **use**	**Symptom assessment**	**Length of treatment**	**Primary outcome time point**	**Total sample size**	**ROB assessment**
Amminger 2010^68^	CHR	PUFA	1.2 g	No	PANSS	12 weeks	52 weeks	81	Low
McGorry 2017^69^	CHR	PUFA	2.8 g	No	BPRS	26 weeks	52 weeks	304	Some concerns
Qurashi 2023^63^	CHR	PUFA and Minocycline	PUFA 1.2 g, Minocycline 200 mg.	No	Transition only	26 weeks	52 weeks	326	Some concerns
Wasserthal 2024^62^	CHR	NAC and psychological intervention	2 g	No	Transition only	26 weeks	52 weeks	46	High
Winter Van-Rossum 2024^55^	CHR	PUFAs	1.2 g	Allowed and used in 3 participants	PANSS	26 weeks	104 weeks	135	Some concerns
Breier 2018^50^	FEP	NAC	3.6 g	Yes (reducing does)	PANSS	52 weeks	52 weeks	60	Some concerns
Deakin 2018^51^	FEP	Minocycline (antibiotic)	300 mg	Yes	PANSS	52 weeks	52 weeks	207	Low
Emsley 2014^70^	FEP	PUFA	2 g	Yes	PANSS	104 weeks	104 weeks	33	Some concerns
Husain 2024^52^	FEP	NAC and Sodium Benzoate	NAC 2 g, Sodium Benzoate 1 g	Yes	PANSS	12 weeks	12 weeks	68	Some concerns
Liu 2018^71^	FEP	Minocycline (antibiotic)	400 mg	Yes	PANSS	16 weeks	16 weeks	55	High
Müller 2010^53^	FEP	Celecoxib	800 mg	Yes	PANSS	6 weeks	6 weeks	50	High
Pawetczyk 2016^72^	FEP	PUFA	2.2 g	Yes	PANSS	26 weeks	26 weeks	71	Low
Pyatoykina 2020^57^	FEP	NAC	2 g	Yes	PANSS	8 weeks	8 weeks	18	Some concerns
Ritsner 2014^73^	FEP	Pregnenolone	50 mg	Yes	PANSS	8 weeks	8 weeks	52	Some concerns
Robinson 2019^54^	FEP	PUFA	1.14 g	Yes	BPRS	16 weeks	16 weeks	50	High
Sommer 2021^74^	FEP	Simvastatin	40 mg	Yes	PANSS	52 weeks	52 weeks	119	Some concerns
Zhang 2021^56^	FEP	Celecoxib	200 mg	Yes	PANSS	6 weeks	6 weeks	93	High

Abbreviations: CHR = clinical high risk, FEP = first episode psychosis, PUFA = polyunsaturated fatty acids, NAC = N-acetylcysteine, PANSS = positive and negative symptom scale, BPRS = brief psychiatric rating scale, ROB = risk of bias.

**Figure 1:**
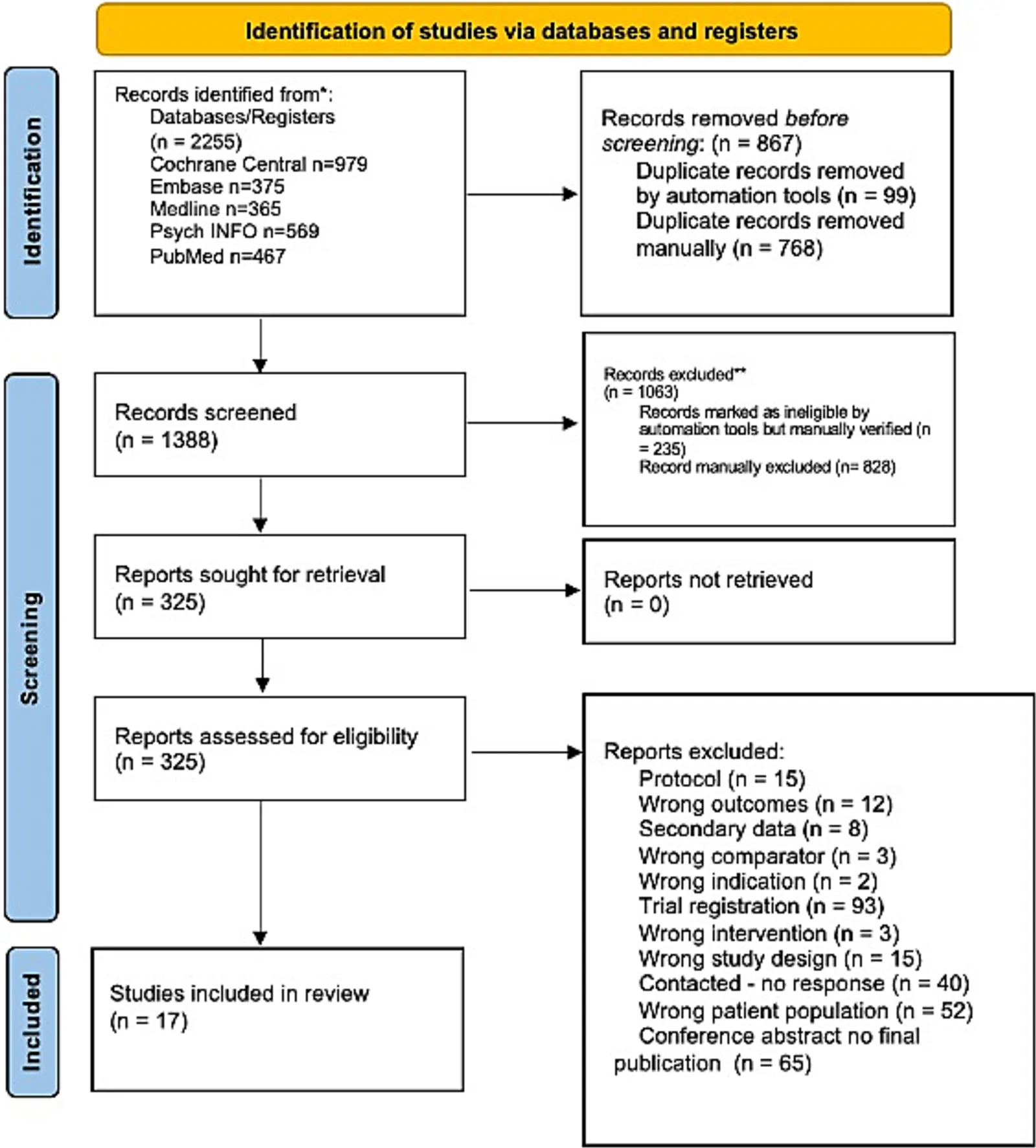
Prisma Flow Diagram.

CHR: 5 studies included a total of *n* = 892 participants. The studies investigated PUFAs (*k* = 4)^55^^,^^63^^,^^67^^,^^69^ and NAC (*k* = 1).^62^ Only one study allowed concurrent psychotropic medication use,^55^ which permitted the use of antipsychotics, antidepressants, and benzodiazepines. Only a minority, 3 out of 68 of those in the placebo arm, were on antipsychotics, and zero in the treatment arm. The duration of treatment varied between 12 and 26 weeks. In 3 studies,^55^^,^^63^^,^^69^ CHR status and transition were defined using the CAARMS,^41^ one^62^ used the SIPS,^42^ and one^68^ used the PANSS assessment with cutoffs of severity and duration defined by outlined by Morrison et al.^75^ and Yung et al.,^76^ accordingly.

FEP: 12 studies included a total of *n* = 876 participants. The studies included NAC (*k* = 3),^50^^,^^52^^,^^57^ with one of these investigating NAC in combination with sodium benzoate,^52^ minocycline (*k* = 2),^51^^,^^71^ PUFAs (*k* = 3),^54^^,^^70^^,^^72^ celecoxib (*k* = 2),^53^^,^^56^ pregnenolone (*k* = 1),^73^ and simvastatin (*k* = 1).^74^ All studies investigated anti-inflammatories as add-ons to concurrent antipsychotic use. One study looked at relapse prevention in the context of antipsychotic discontinuation.^70^ Duration of treatment varied between 6 weeks and 2 years. See Table 1 with study reference and further information.

One additional study was found to meet the inclusion criteria^77^; however, the paper did not have the outcome data report required. No response was received following a request for this information. See the supplementary Section 9 for further information.

### Risk of Bias Assessment

One CHR study,^68^ and two FEP studies,^51^^,^^72^ had low risk for all assessment domains. All other studies had either some concerns or a high risk of bias in at least 1 of the 5 domains. One CHR,^62^ and four FEP studies^53^^,^^54^^,^^56^^,^^71^ were assessed as having an overall high risk of bias. The most common area impacting ROB was missing data. For the results of the ROB assessment, please see Supplementary Section 10 and Supplementary Table S1.

### Primary Outcome Results

CHR: meta-analysis of 5 studies (*n* = 892) found no significant effect of anti-inflammatory treatments on the risk of transition to psychosis compared with placebo (OR = 0.88, 95% CI, 0.26-3.01, *P* = .75, *I*^2^ = 59%), forest plot in Figure 2.

**Figure 2:**
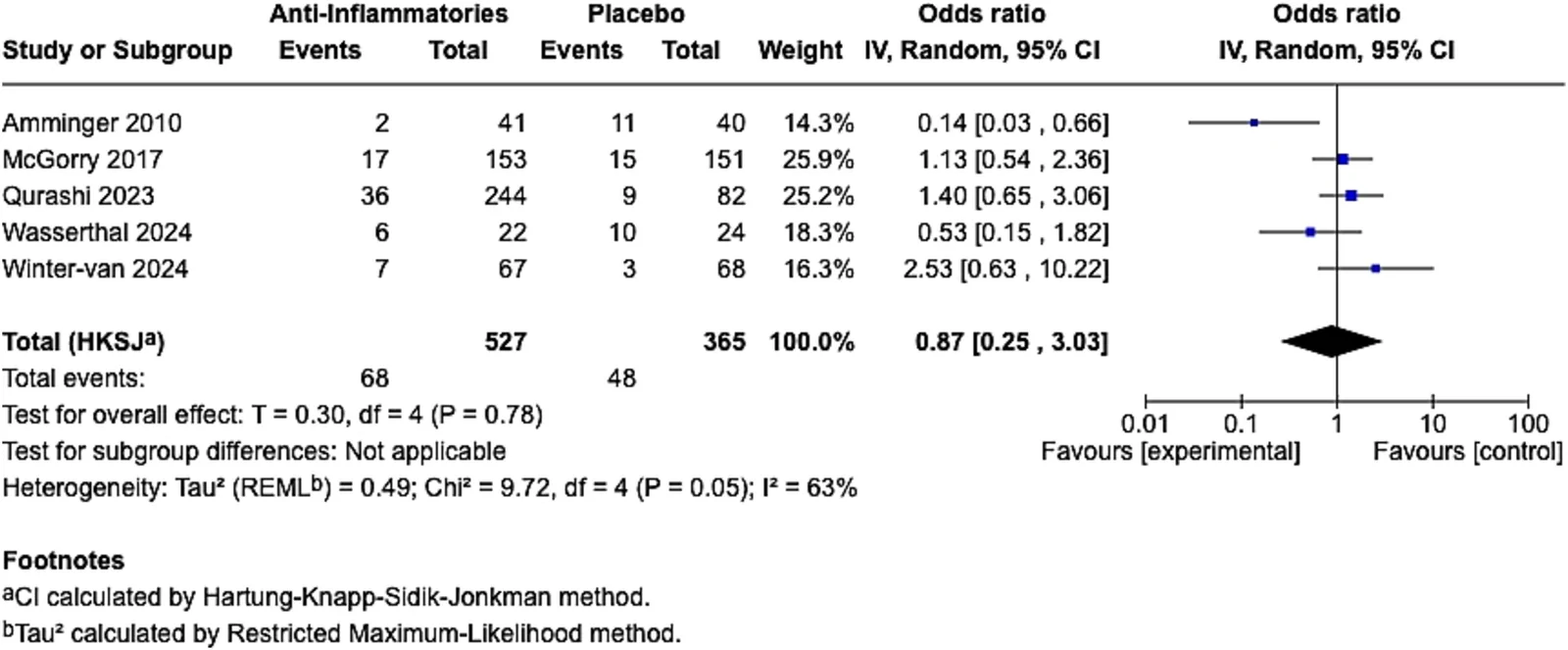
Risk of Transition to Psychosis in CHR.

FEP: meta-analysis of 12 studies (*n* = 876) found that anti-inflammatory treatments significantly reduced psychotic symptoms compared to placebo (SMD = −0.38, 95% CI, −0.76 to 0.00, *P* = .05, *I*^2^ = 81%), see forest plot in Figure 3.

**Figure 3:**
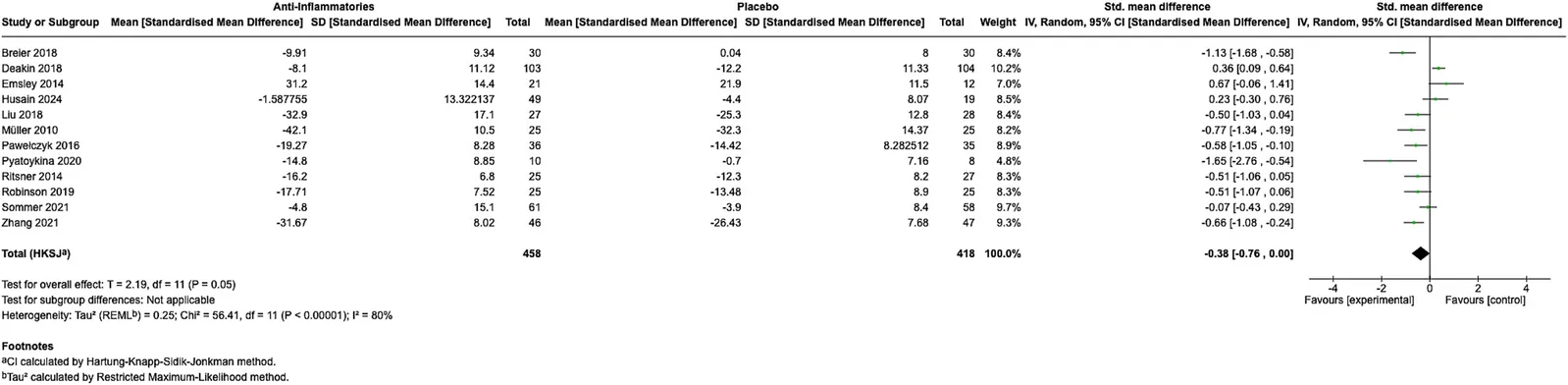
Change in Psychotic Symptoms in FEP.

### Secondary Outcomes

CHR: in the three studies reporting symptom scores,^55^^,^^68^^,^^69^ anti-inflammatory treatments had no effect on psychotic symptom changes compared to placebo (SMD = 0.00, 95% CI, −1.49 to 1.50, *P* = .99), see Supplementary Figure S1.

FEP: 11 studies assessed the effect of anti-inflammatory treatments on PANSS symptom sub-scores. We found a small but statistically significant reduction in PANSS positive symptoms (MD = −0.92, 95% CI, −1.80 to −0.04, *P* = .04, *I*^2^ = 55%). However, reductions in PANSS negative (MD = −0.69, 95% CI, −2.42 to 1.04, *P* = .40, *I*^2^ = 84%) and PANSS general symptoms (MD = −0.19, 95% CI: −1.97 to 1.59, *P* = .82, *I*^2^ = 71%) were not statistically significant. No significant reduction was observed for PANSS Total scores (MD = −3.61, 95% CI, −8.01 to 0.79, *P* < .10, *I*^2^ = 83%). In contrast to our primary outcome in FEP, this result is not significant. This may be because one study^54^ measured BPRS rather than PANSS and could not be included in this analysis (Supplementary Figures S2-S5 for forest plots).

Subgroup analysis compared classical and non-classical anti-inflammatory treatments. Classical anti-inflammatories (which pooled results from trials of minocycline and celecoxib) showed no significant effect on psychotic symptoms (SMD = −0.37, 95% CI, −1.21 to 0.48, *P* = .25, *I*^2^ = 88%, *k* = 4, *n* = 405). Non-classical anti-inflammatories (which pooled results from trials of NAC, PUFAs, sodium benzoate, pregnenolone, and a statin) also showed no significant effect on psychotic symptoms (SMD = −0.39, 95% CI, −0.95 to 0.17, *P* = .14, *I*^2^ = 76%, *k* = 8, *n* = 471), with high heterogeneity present in both subgroups (Supplementary Figure S6 for forest plot). This was not possible for the CHR group, as exclusively non-classical antipsychotics were used.

We also examined individual classes of anti-inflammatory treatments. Many of the classes only had one study for each class of anti-inflammatory, with the exception of PUFAs, NAC, celecoxib, and minocycline. Celecoxib (SMD = −0.70, 95% CI, −1.33 to −0.07, *P* = .05, *I*^2^ = 0%, *k* = 2, *n* = 143) demonstrated significant improvement in symptoms. PUFAs (SMD = −0.18, 95% CI, −1.88 to 1.51, *P* = .29, *I*^2^ = 77%, *k* = 3, *n* = 154), NAC (SMD = −0.79, 95% CI, −3.19 to 1.61, *P* = .17, *I*^2^ = 88%, *k* = 3, *n* = 146), and minocycline (SMD = −0.03, 95% CI, −5.59 to 5.42, *P* = .94, *I*^2^ = 87%, *k* = 2, *n* = 262) showed no significant improvements in symptoms (Supplementary Figure S7 for forest plots).

One study, Emsley et al.^70^ investigated the effect of a PUFA in relapse prevention in those having anti-psychotic medication withdrawn. PANSS scores in all domains increased in both intervention and placebo groups, reflecting a worsening of symptoms after withdrawal of antipsychotic medication, with no significant difference in relapse symptom severity between groups. When this study is excluded, results remain significant with a larger effect size (SMD = −0.45, 95% CI, −0.81 to −0.09, *P* = .02, *I*^2^ = 77%) (Supplementary Figure S8).

### Sensitivity Analyses

As predicted, levels of heterogeneity were generally high, so a random-effects model was used. As a sensitivity analysis, fixed-effect models were used for comparison for all the primary outcomes. For FEP, the result remained significant but with a smaller effect size for change in psychotic symptoms (SMD = −0.23, 95% CI, −0.37 to −0.09, *P* = .0009, *I*^2^ = 81%). For CHR, the result remained unchanged, with no significant effect of anti-inflammatory treatments on the risk of transition to psychosis compared with placebo (OR = 1.01, 95% CI, 0.65-1.57, *P* = .97, *I*^2^ = 59%) (Supplementary Figures S9 and S10 for forest plots).

For the FEP primary outcome, heterogeneity was high (*I*^2^ = 80%), indicating significant variance between studies (Tau^2^ = 0.25). Meta-regression analysis did not show a significant impact of ROB assessment (*Q* = 1.56, *P* = .46). However, studies with high ROB (*k* = 4) showed the largest treatment effects (SMD = −0.61, 95% CI, −0.81 to −0.42), while those with low (*k* = 2) showed no significant treatment effect (SMD = −0.09, 95% CI, −6.207 to 5.90). For FEP, when we exclude studies with a high ROB, the effect size is smaller, and the results are no longer significant (SMD = −0.28, 95% CI, −0.89 to 0.34), raising concerns about low-quality studies overestimating effect sizes. For CHR, the non-significant results remain. Sample size was a significant moderator (*QM* = 13.94, *P* = .0002), accounting for nearly three-quarters of the heterogeneity (*R*^2^ = 74.24%). The regression coefficient was positive (β = 0.0067, *P* = .002), indicating that studies with larger sample sizes tended to report smaller effect sizes. Residual heterogeneity was non-significant after accounting for sample size (*I*^2^ = 40.15%, QE = 16.95, *P* = .076). When assessing the individual types of anti-inflammatories used, we found significant subgroup differences (Q = 15.18, *P* = .0145). However, as several subgroups contained only a single study, these findings should be interpreted with caution due to the risk of study-level bias. Other factors, such as classical vs non-classical anti-inflammatory, year of publication, duration of treatment, and mean age of participants, did not significantly explain the heterogeneity. In short, both sample size and the range of anti-inflammatories used may have impacted the high levels of heterogeneity. The results of the leave-one-out analysis showed that for CHR, the effect estimates remained stable across all iterations, suggesting a more robust overall result. For FEP, the statistical significance of the pooled effect varied with the exclusion of individual studies. Specifically, the removal of any single study favoring the intervention arm led to a loss of statistical significance. One exception was Sommer et al.,^74^ which favored the intervention (though not significantly). When this study was removed, the overall meta-analysis remained significant. This suggests the findings for FEP may not be as robust, so caution should be taken when interpreting these results (Supplementary Tables S2 and S3). For results of any other sensitivity analyses, please refer to the Supplementary Section 14.

For the CHR meta-analysis, Eggers test, which should be interpreted with caution in a sample of only five studies, showed no statistically significant evidence of small-study effects (*t* = −1.04, df = 3, *P* = .3735) (Supplementary Figure S12 for funnel plot).

For FEP, a funnel plot and Eggers test indicate some possible asymmetry, but not statistically significant (*t* = −2.20, df = 10, *P* = .052) (Supplementary Figure S13). The trim-and-fill analysis estimated that one small study with negative results may be missing from the right side of the funnel plot (Supplementary Figure S14), likely leading to an overestimation of the treatment effect. After adjusting for the missing study, the effect size was reduced from −0.38 to −0.32 and was no longer statistically significant (SMD = −0.32, 95% CI: −0.65 to 0.019, *P* = .064, *I*^2^ = 81%).

While some individual studies showed evidence of variability between intervention and control groups,^52^^,^^74^ comparisons of SDs and coefficients of variation did not show a significance in pooled results (Supplementary Figures S15 and S16).

Certainty of evidence was assessed as being very low for rates of transition in CHR and changes in symptoms in FEP, primarily due to the non-significant result for CHR, the high heterogeneity in the results and the impact of ROB on the primary results. For the full assessment and reasoning, see the GRADE assessment in the Supplementary Section 14.

## Discussion

This article represents the first systematic review and meta-analysis of anti-inflammatory interventions focusing specifically on the early stages of psychosis across CHR and FEP. For CHR, our summary evidence suggests no significant effect of adjunctive anti-inflammatories on rates of transition to psychosis and no effect on symptom measures in this group. On balance, our results imply that those presenting below a clinically significant threshold for psychosis may not benefit from anti-inflammatory treatment. Several factors may underlie the non-significant findings in the CHR subgroup, notably issues of power and heterogeneity. First, a substantial proportion of individuals identified as CHR do not go on to develop psychosis, making it difficult to detect significant between-group differences without very large sample sizes. Although the pooled sample was large (*n* = 892), results may still have been underpowered. Second, CHR populations are highly heterogeneous, not only in outcomes but in underlying mechanisms. If anti-inflammatory treatments are only likely to be effective in those with both elevated inflammation and true risk of transition, effects may be diluted in general CHR cohorts.

In FEP, results demonstrate a small but significant improvement in symptoms following anti-inflammatory treatment. When examining the impact on specific sub-scores and types of symptoms, significant results were found for total psychotic symptom severity scores and PANSS positive scores, but not for negative and general symptoms individually.

These findings provide further evidence for the potential role of anti-inflammatory treatments in psychotic disorders, emphasizing that their effect may be increased in early stages. Two meta-analyses^26^^,^^27^ have investigated the use of adjunctive anti-inflammatory medications in all stages of psychosis (FEP through to chronic). Jeppesen et al.^26^ also found a significant effect for add-on anti-inflammatories with an effect size (SMD = −0.29, 95% CI, −0.40 to 95%0.19). However, when this meta-analysis looked at the effect in earlier stages of psychosis, the effect was lost for those with a duration of less than 2 years. In our analysis, looking at those with a mean of 3 years or less, it was significant. This is likely to be influenced by the larger number of studies included in our review, with 12 studies, compared to the 5 in the Jeppesen et al.^26^ meta-analysis.

We detected significant improvement only in total and positive symptom sub-scores, which contrasts with the findings of Jeppesen et al., where significant improvement was observed across all PANSS subscales. Previous research has linked negative symptoms in psychosis to inflammation,^11^ leading to the hypothesis that they might be the most likely to respond to anti-inflammatory treatment, though this was not the case in our FEP sample. However, our sample was limited to FEP studies as opposed to the larger chronic sample in the Jeppesen 2020 meta-analysis. Furthermore, negative symptoms may become more prominent in the later stages of psychotic disorders,^78^^,^^79^ which may influence or limit their responsiveness to treatment in early-stage populations.

Previous reviews have also compared classical vs nonclassical anti-inflammatory interventions in all stages of schizophrenia, concluding that both classes of interventions were effective, with no superiority of either. In contrast, in this study, we have found no significant effects for either classical or non-classical subgroups. This may, in part, be due to reduced power when dividing the sample of 12 studies into smaller subgroups. The lack of significance in both subgroups does not necessarily imply the absence of effect but highlights the limitations of interpreting subgroup findings in the context of limited data. These findings should therefore be interpreted with caution, as the analyses may be underpowered to detect meaningful differences between intervention classes. When looking at individual anti-inflammatory classes, we found significant results for celecoxib. This mirrors previous results for celecoxib, where a meta-analysis from 2017 found a positive effect of celecoxib only in studies with predominantly FEP participants.^34^ Jeppesen et al. also found significant results for PUFAs, NAC, and antibiotics, again benefitting from larger sample sizes due to more inclusive criteria. Another review from 2019,^27^ looking at all stages of illness, containing many of the same studies, found significant results for NAC and minocycline, but not PUFAs. Interestingly, this study ran a sensitivity analysis looking at only FEP studies, none of which were significant. In our review, we captured more studies than in these sensitivity analyses.

Given the hypothesis that inflammation contributes to psychosis in only a subset of individuals,^20^ we examined variance in treatment effects to investigate potential heterogeneity in treatment response, specifically, whether individuals with inflammation may respond differently to anti-inflammatory treatment compared to those without inflammation. While some individual studies showed variability in response,^52^^,^^74^ the pooled results did not reflect a consistent pattern.

Regarding future research, future trials should prioritize more targeted approaches. In the CHR population, where transition rates are low and heterogeneity is high, stratification may first be needed to define the cohort for which anti-inflammatories should be targeted. The use of inflammatory biomarkers may aid this endeavor. Furthermore, consideration should be given as to whether transition to psychosis is the most relevant primary outcome, or if other common outcomes in the CHR group, such as depression or anxiety, may also be relevant targets.

In FEP, where transition has already occurred, biomarker-based stratification may also help clarify whether anti-inflammatory treatments are effective in specific subgroups rather than across the broader psychosis population. Promising early examples include the PIMS study,^80^ which stratifies participants by IL-6 levels at enrolment, enrolling only those with elevated IL-6 into the treatment arm. Further work is needed to identify optimal biomarkers, define clinically meaningful cut-offs, and determine how best to combine them with other phenotypic indicators such as neuroimaging or clinical profiles. Deep phenotyping of psychosis may help refine the definition of inflammatory subtypes and improve the precision of future stratified trials.^81^ Ethical considerations are also essential, as inaccurate stratification could risk excluding individuals who might otherwise benefit from treatment.

### Strengths and Limitations

This is the first systematic review and meta-analysis specifically examining the use of anti-inflammatory treatments in the early stages of psychosis (FEP and CHR). We believe that this focus is crucial, as patients in these early stages present with distinct symptom profiles and clinical characteristics. By focusing specifically on studies of these early psychosis subgroups, our analysis improves the interpretation of findings for clinical practice and helps guide future research directions. Additionally, we have highlighted more recent studies; whereby, of the 17 included in our review, 6 have been published since the largest previous systematic review and meta-analysis on anti-inflammatories in psychotic disorders. Finally, rigorous assessment of study data bias using the ROB2 tool and certainty of evidence using the GRADE tool, along with detailed sensitivity analysis, helps to provide insight and robustness to our results.

However, several factors warrant cautious interpretation of these results. Several included studies have relatively small sample sizes, with meta-regression and sensitivity analysis suggesting a small study effect, where larger studies were more likely to demonstrate a smaller effect size. Many analyses showed substantial heterogeneity. The generally high ROB across studies is also a limitation. For FEP, studies at lower risk of bias showed smaller and less precise effects, with wide confidence intervals, while those at high risk reported larger, significant effects. When high-risk studies were excluded, the overall effect size was reduced and no longer significant. This raises concerns that lower-quality studies may be inflating the perceived efficacy of the intervention. These results underscore the need for high-quality, low-bias trials to more accurately estimate treatment effects. Furthermore, for FEP, there was an indication of publication bias, suggesting the possibility of unpublished negative studies. We were unable to quantitatively investigate the effect of interventions on inflammation as most studies did not assess or report inflammatory levels or markers. Furthermore, the leave-one-out analysis indicated a degree of fragility in the overall results, with 7 of the 12 studies having a clear influence on the observed result. Most studies focused on shorter-term interventions and short- to medium-term outcomes, limiting conclusions about the sustained efficacy of anti-inflammatory treatments over time. Some studies were excluded despite potentially containing data on people meeting the criteria for FEP. Had we been able to acquire individual patient data instead of aggregate data, we may have been able to include a wider range of participants, studies, and interventions. Overall findings of our meta-analysis should be interpreted with some caution.

## Conclusion

This meta-analysis found significant positive effects of anti-inflammatory treatments in FEP, suggesting a potential therapeutic benefit in this population. However, there was no clear evidence that anti-inflammatory interventions reduce the risk of transition to psychosis from CHR. Studies were likely underpowered to detect a significant difference in symptoms in CHR. Future research should focus on addressing key methodological limitations observed in the existing literature. Large, well-designed studies are needed to mitigate the impact of small-study effects and ensure robust conclusions. Studies should prioritize rigorous methodologies that minimize the risk of bias, particularly regarding missing data. Finally, future trials should consider incorporating biomarker-based stratification to identify and target those with elevated inflammatory markers.

## Supplementary Material

Supplementary_Materials_revision_clean_final_sbaf173
