# Supplementary material for: Systematic Review and Meta-Analysis of Randomized Clinical Trials of Anti-Inflammatory Agents in Early-Stage Psychotic Disorders
Source: Schizophr Bull. 2025 Oct 28;52(5):sbaf173. doi: 10.1093/schbul/sbaf173 (PMC13645840; doi:10.1093/schbul/sbaf173)
Supplement: Supplementary file 1 [file supplementary_materials_revision_clean_final_sbaf173.doc]

**Supplementary materials**

**Contents**

Supplementary methods

1. Protocol and amendments
2. Search strategy
3. Request to authors
4. Handling of missing data
5. Multiple-arm intervention studies
6. Handling of Different Symptom Rating Scales
7. Sensitivity analysis methods
8. GRADE assessment

Supplementary results

9. Other identified studies
10. Risk of Bias assessment results
11. Secondary outcomes
    a. Symptom changes in CHR
    b. PANSS symptom sub-scale outcomes
12. Results for different types of anti-inflammatories
    a. Classical vs Non-classical
    b. Individual types of anti-inflammatories
13. Exclusion of Emsley 2014.
14. Sensitivity analyses
    a. Fixed-effect models for primary outcomes
    b. Exploration of heterogeneity with meta-regression
    c. Leave-one-out analyses
    d. Effect of study size
    e. Durartion of Illness less than 2 years
    f. Variation of means and coefficient results
15. Grade assessment results

**Supplementary methods**

**Section 1. Protocol and amendments**

The study protocol was registered in Prospero (ID CRD42020226925) in December 2020 when the review was conducted.

Minor amendments to the protocol include the following. In the protocol, we state, “The primary outcome will be changes in positive and negative symptoms in individual patients using scores such as the Positive and Negative Syndrome Scale, the Brief Psychiatric Rating or clinical global impression before and after intervention.” In the study, the primary outcome for First episode psychosis (FEP) was the change in total symptom scores, with changes in sub-scores as a secondary outcome. For clinical high risk (CHR) in the protocol we state “categorical data to be compared using risk ratios”, the study we used odds ratios to asses rates of transition. A full copy of the review protocol can be found at <https://www.crd.york.ac.uk/PROSPERO/view/CRD42020226925>.

**Section 2. Search strategy**

Broadly, the search strategy combined terms relating to first-episode psychosis or clinical high risk for psychosis with terms relating to anti-inflammatory medications, either generally or using named examples, with terms attempting to identify randomised controlled trials. The primary search was conducted on Wednesday 22^nd^ November 2023. An example search strategy, used to search EMBASE (Ovid) is as follows:

(((FEP OR psychosis OR psychoses OR psychotic OR schizophreni* OR schizophreniform OR schizoaffective) OR ("ultra high risk of schizophrenia" OR "ultra high risk of psychosis" OR "at risk mental state for schizophrenia" OR "at risk mental state for psychosis" OR "high risk for schizophrenia" OR "high risk for psychosis" OR "prodrome of schizophrenia" OR "prodrome of psychosis" OR "genetic high risk for schizophrenia" OR "genetic high risk for psychosis" OR "familial high risk for schizophrenia" OR "familial high risk for psychosis" OR "unaffected relatives of schizophrenia probands" OR "unaffected relatives of psychosis probands" OR "clinical high risk of schizophrenia" OR "clinical high risk of psychosis"))

AND

(anti-inflammatory OR "non-steroidal anti-inflammatory" OR "acetylsalicylic acid" OR aspirin OR NSAID OR ibuprofen OR diclofenac OR indomethacin OR "cyclooxygenase 2 inhibitor" OR cox-2 OR celecoxib OR "N-acetyl cysteine" OR NAC OR statins OR pravastatin OR simvastatin OR atorvastatin OR corticosteroids OR glucocorticoids OR pregnenolone OR prednisone OR prednisolone OR hydrocortisone OR methylprednisolone OR dexamethasone OR cortisone OR minocycline OR tetracyclines OR "monoclonal antibodies" OR rituximab OR infliximab OR etanercept OR tocilizumab OR estrogen OR raloxifene OR tamoxifen OR EPA OR DHA OR PUFA OR "poly-unsaturated fatty acids" OR "omega-3" OR melatonin OR methotrexate OR azathioprine OR "intravenous immunoglobulin" OR IVIG OR immunosuppression OR "immunosuppressive agents")

AND

(RCT OR randomised OR randomized OR randomly OR "randomized controlled trial" OR "randomised controlled trial" OR "randomized control trial" OR "randomised control trial" OR placebo)).ab,ti

This same search was used for each database, with the exact search undergoing only syntactic modification to facilitate each database search.

The supplementary search was conducted using the identical, previously written search terms and was conducted on 6^th^ January 2025.

**Section 3. Request to authors**

Where clarification was required on any aspect of the studies at either point of the intervention assessment or data extraction, authors were contacted. When deciding if a search result was eligible for inclusion, there were three main reasons authors were contacted. Firstly, if a conference abstract was found, to ask if the results had either been published or if the results were otherwise available. Secondly, if a trial registration was found, if the trial was completed, and data was either published or was available to share. Thirdly, if the duration of illness was not clear, or if the sample could reasonably contain people within the CHR or FEP populations, authors were contacted to confirm the duration of illness criteria for the study, the mean duration of illness for the sample, or if anonymised individual data for those meeting our criteria could be isolated and shared. We asked for confirmation of receipt of the email be given with the intent to reply within 14 days of the email being sent.

**Section 4. Handling of Missing data**

Our primary effect measure was the mean difference (i.e. change in mean from baseline to primary follow-up) along with the corresponding standard deviation (SD). This was applied across all outcomes, including total PANSS, as well as the PANSS-positive, PANSS-negative, and PANSS-general subscales. However, there was considerable variation in how data were reported across studies. In many cases, the mean change and its SD were not reported directly. Where this occurred, we calculated the mean difference manually using the reported means and SDs at baseline and follow-up, in accordance with guidance from chapter 6 of the Cochrane Handbook.^2^

Missing SDs

Where standard error (SE) was reported instead of standard deviation (SD), the SD was calculated using the following formula:

SD = SE × √(n),

where n is the sample size.

This conversion was applied to six studies: Amminger (2010),^3^ Breier (2018),^4^ Deakin (2018),^5^ Pawełczyk (2016),^6^ Pyatoykina (2020),^7^ and Robinson (2019).^8^

Missing mean difference and corresponding SDs

Where only baseline and follow-up means and standard deviations (SDs) were reported, we calculated the mean difference and corresponding SDs following the method outlined in the Cochrane Handbook. This approach was applied to seven studies: Breier (2018),^4^ Deakin (2018),^5^ Husain (2024),^9^ Müller (2010),^10^ Robinson (2019),^8^ Winter van Rossum (2024),^11^ and Zhang (2021).^12^

The mean difference was calculated by subtracting the baseline mean from the follow-up mean for each group:

The standard deviation of the mean change between baseline and follow-up (SD_chnage_) was calculated using the following formula:

Here, “corr” refers to the correlation coefficient, which reflects the relationship between baseline and follow-up scores within individuals. This value is not typically reported in primary studies and differs between studies and outcomes. However, it can be estimated from another study that reports means and SDs for baseline, follow-up, and change scores for the same outcome. The correlation coefficient was calculated separately for each PANSS subscale (total, positive, negative, and general) using the following formula:

We calculated the correlation coefficient using data from studies that reported complete information, including baseline, follow-up, and change scores. These calculated coefficients were then used to impute values for studies that did not report sufficient data, selecting the most appropriate match based on the type of intervention and the size of the study.

For studies that used non-classical anti-inflammatory treatments — specifically Breier (2018),^4^ Winter van Rossum (2024),^11^ and Husain (2024)^9^ — we used the correlation coefficient derived from Pawełczyk (2016).^6^ For studies involving classical anti-inflammatories, such as Deakin (2018),^5^ Müller (2010),^10^ and Zhang (2021),^12^ we used the coefficient from Ritzner (2014).^13^

In the case of studies reporting BPRS outcomes, McGorry (2017)^14^ was the only study with complete data available. As such, its correlation coefficient was used to inform calculations for Robinson (2019).^8^

Missing total or sub-scores

Some studies did not report data for all PANSS subscales or for the total PANSS score. The PANSS consists of three subscales: PANSS(positive), PANSS(negative), and PANSS(general), which can be summed to produce a PANSS(total) score:

In Pyatoykina (2020),^7^ the authors reported mean scores and standard deviations (SDs) for the three subscales but did not provide values for the total score. To address this, we calculated the total mean by summing the subscale means:

The SD of the total score was then calculated under the assumption that the three subscales were uncorrelated. This was because the raw scores would have been needed to test for correlation; it was not possible with the mean scores alone. Assuming scores were uncorrelated gives a more conservative estimate. The formula used was:

This method was applied to derive PANSS(total) at baseline, follow-up, and for the change between them.

In Deakin (2018),^5^ the study reported the mean and SD for PANSS(total), PANSS(positive), and PANSS(negative) at both baseline and follow-up, but did not report PANSS(general). We calculated the mean for PANSS(general) by subtracting the positive and negative subscale means from the total score:

This was done for baseline, follow-up, and change scores.

To calculate the standard deviation (SD) of the PANSS(general) change score, we considered two approaches. One involved deriving it using the reported SDs for the change in mean for PANSS(total), PANSS(positive), and PANSS(negative). The other approach involved calculating it from the SDs of PANSS(general) at baseline and follow-up. We found that the second method provided a more conservative estimate, and therefore used this value in our analysis.

**Section 5. Multiple-arm intervention studies**

There were 3 studies that included more than a single intervention. Husain 2024 compares both NAC and Sodium Benzoate in a 4-arm study (double placebo, vs NAC and placebo, vs Sodium Benzoate and placebo vs NAC and Sodium Benzoate). For this study, all of those who received an anti-inflammatory were combined into one group for comparison to placebo as recommended by the Cochrane handbook chapter 23,^1^ as our primary outcome was looking at the effect of any anti-inflammatories.

Qurashi 2023 compares both a PUFA and Minocycline again in a 4-arm study (double placebo, vs PUFA and placebo, vs Minocycline and placebo vs PUFA and Minocycline). Similarly to Husain 2024, all of those who received an anti-inflammatory were combined into one group for comparison to the placebo. Both have been excluded from sensitivity analyses which look at individual types of anti-inflammatories.

Finally, Wasserthal 2024 compares NAC alongside a psychological intervention, integrated preventive psychological intervention (IPPI) which has a placebo intervention, psychological stress management (PSM), a form of psychoeducation, again in a 4 arm study (placebo and PSM, vs NAC and PSM, vs placebo and IPPI, vs NAC and IPPI). For this study, all arms were included, as roughly half received NAC and either IPPI or PSM, and the other half receiving placebo and either IPPI or PSM, by comparing those of received NAC vs placebo over all this was likely to represent a well match comparison.

**Section 6. Handling of Different Symptom Rating Scales**

In our primary meta-analysis of symptom change in first-episode psychosis (FEP), studies employed different psychometric scales to assess psychotic symptoms, most commonly the Positive and Negative Syndrome Scale (PANSS) and the Brief Psychiatric Rating Scale (BPRS). To account for these differences, we used standardised mean differences (SMD), specifically bias-corrected Hedges’ g, to pool effect sizes. This method enables the combination of continuous outcomes measured on different scales by expressing them as a function of standard deviation units.

Both PANSS and BPRS are widely used and validated measures of general psychosis severity. While PANSS includes positive, negative, and general psychopathology subscales, we extracted total scores to align with the structure of BPRS, which does not routinely report subscale scores in trial data. Using total scores across both scales improves consistency and comparability.

The SMD calculations were performed using Cochrane Review Manager (RevMan, Version 8.14), which computes SMDs from group means, standard deviations, and sample sizes. Where only change scores were reported, these were used; otherwise, we used post-treatment scores. This method aligns with recommendations from the Cochrane Handbook and is consistent with previous meta-analyses in psychosis populations that have pooled different symptom rating scales.

**Section 7. Sensitivity Analysis Methods**

Several sensitivity analyses were conducted to assess the robustness of the primary findings and explore potential sources of heterogeneity.

Meta regression

Meta-regression analyses were performed to investigate whether study-level characteristics—including sample size, year of publication, risk of bias, and class of anti-inflammatory—explained variability and heterogeneity across studies. All meta-regressions were performed in RStudio using the metafor package (version 4.8.0), employing a random-effects model with the Hartung-Knapp adjustment. Continuous variables (e.g. year, sample size) were entered as moderators, and categorical variables (e.g. ROB, intervention type) were entered as factors.

Leave-one-out analysis

To evaluate the robustness of the meta-analysis results and identify any individual studies that might disproportionately influence the overall findings, we conducted a leave-one-out sensitivity analysis. This involved systematically removing one study at a time and recalculating the pooled effect size for the remaining studies. By comparing these recalculated effect sizes, we assessed whether the overall result was stable or heavily dependent on any single study. If the pooled effect size remained consistent across all iterations, it suggests that the findings are robust. Conversely, significant changes in the effect size after excluding a particular study indicate that the meta-analysis results may be sensitive to that study’s inclusion, warranting cautious interpretation.

Publication bias and small study effects

To evaluate the possibility of publication bias and small study effects, we used multiple complementary methods. Funnel plots were visually inspected to assess the symmetry of study effect sizes around the pooled estimate. Egger’s regression test was performed to statistically detect funnel plot asymmetry, with a significant result indicating possible small study effects or publication bias. To further explore and adjust for potential missing studies due to publication bias, we applied the trim-and-fill method. This method estimates the number and effect sizes of potentially missing studies to provide an adjusted pooled effect size, offering a more conservative estimate that accounts for bias. Together, these analyses help assess whether the meta-analysis results may be influenced by selective reporting or other biases related to study size. These sensitivity analyses were conducted in RStudio using the metafor package (version 4.8.0).

Variability of outcome measure

To explore whether the variability in treatment response differed between intervention and control groups, we conducted sensitivity analyses using two complementary approaches: standard deviation (SD) ratios and coefficient of variation (CV) ratios. These methods aim to assess whether some individuals may experience much stronger effects than others, which can indicate underlying heterogeneity in response to the intervention.

For the SD ratio analysis, we first extracted the means and standard deviations (SDs) for both treatment and control groups from each study. If only standard errors (SEs) were reported, we converted these to SDs using the formula: SD = SE × √n, where n is the group size. Within each study, we then calculated the ratio of SDs by dividing the SD of the treatment group by that of the control group. A ratio greater than 1 suggests greater variability in the treatment arm. Because SD ratios tend to be skewed, we log-transformed the ratios before pooling them across studies using a random-effects meta-analysis.

Similarly, we calculated the coefficient of variation (CV) for each group in each study by dividing the SD by the group mean. This provides a measure of relative variability, accounting for differences in outcome magnitude. We then computed the CV ratio by dividing the CV of the treatment group by that of the control group. As with the SD ratios, CV ratios were log-transformed and pooled using a random-effects meta-analysis.

Together, these analyses allowed us to examine whether the intervention was associated with increased or decreased variability in outcomes. An increase in variability could suggest a differential treatment response within the intervention group — for example, that a subset of participants may benefit significantly while others show little or no response. This pattern might indicate underlying heterogeneity in treatment efficacy, potentially reflecting meaningful biological or clinical subgroups (e.g., individuals with inflammatory psychosis) who respond more strongly. Such differences could help explain why an overall group effect is not observed, despite some individuals responding well. These sensitivity analyses were conducted in RStudio using the metafor package (version 4.8.0).

**Section 8. GRADE assessment**

The certainty of evidence for each outcome was assessed using the Grading of Recommendations Assessment, Development and Evaluation (GRADE) approach.^15^ GRADE is a widely endorsed framework for evaluating the quality of evidence in systematic reviews and clinical guidelines. It provides a transparent and structured process for rating the certainty of evidence across outcomes, based on a set of predefined domains.^15^

Under this approach, the certainty of evidence is rated as high, moderate, low, or very low. Randomised controlled trials begin as high-certainty evidence but may be downgraded based on five key domains: risk of bias, inconsistency, indirectness, imprecision, and publication bias. Additional factors such as a large magnitude of effect, evidence of a dose–response relationship, or plausible residual confounding may increase the certainty rating in some cases.

GRADE enables reviewers to make consistent and transparent judgments about the confidence in effect estimates, supporting more reliable interpretation and application of findings. Results of the GRADE assessment are in section 14 of this supplementary materials.

**Supplement Results**

**Section 9. Other identified studies**

During the search process, we identified one study, Conus 2018^16^, that initially appeared to meet the inclusion criteria. The study investigated the use of N-acetylcysteine (NAC) in early psychosis. However, neither the main manuscript nor the supplementary materials reported the baseline and follow-up PANSS scores required to calculate our primary outcome.

Interestingly, this study was included in the Jeppesen 2020 review ^17^, so we contacted the corresponding author to request either (a) the PANSS change scores with standard deviations, (b) the baseline and follow-up data so we could calculate the change manually, or (c) confirmation of the values used in the Jeppesen 2020 review. We did not receive a response. As we were unable to verify the data, we chose to exclude the study rather than rely on potentially unconfirmed figures. Notably, the original report found no benefit of the intervention over placebo.

**Section 10. Risk of bias (ROB) assessment and results**

S.Table 1. Summary table of ROB assessment with results for each domain assessed.

| Study Name | Domain 1. Randomization process | Domain 2. Deviations from intended interventions | Domain 3. Missing outcome data | Domain 4. Measurement of the outcome | Domain 5. Selection of the reported result | Domain 6. Overall Bias |
| --- | --- | --- | --- | --- | --- | --- |
| Amminger 2010 | Low | Low | Low | Low | Low | Low |
| McGorry 2017 | Low | Low | Some concerns | Low | Low | Some concerns |
| Qurashi 2023 | Low | Low | Some concerns | Low | Low | Some concerns |
| Wasserthal 2024 | Low | Low | High | Low | Low | High |
| Winter Van-Rossum 2024 | Low | Low | Low | Low | Some Concerns | Some Concerns |
|  |  |  |  |  |  |  |
| Breier 2018 | Some concerns | Some concerns | Some concerns | Low | Low | Some concerns |
| Deakin 2018 | Low | Low | Low | Low | Low | Low |
| Emsley 2014 | Low | Low | Low | Some concerns | Some concerns | Some concerns |
| Husain 2024 | Low | Low | Some concerns | Low | Some concerns | Some concerns |
| Liu 2018 | High | High | High | Some concerns | Some concerns | High |
| Muller 2010 | Low | High | High | Low | Some concerns | High |
| Pawetczyk 2016 | Low | Low | Low | Low | Low | Low |
| Pyatoykina 2020 | Low | Some concerns | Some concerns | Low | Low | Some concerns |
| Ritsner 2014 | Low | Low | Some concerns | Low | Some concerns | Some concerns |
| Robinson 2019 | Low | Low | High | Low | Some Concerns | High |
| Sommer 2021 | Low | Low | Some Concerns | Low | Low | Some Concerns |
| Zhang 2021 | Low | High | Some Concerns | Low | Low | High |

Please refer to the Excel sheet entitled ‘Final_ROB2_IRPG_beta_v9’, also included in the supplementary materials, for the full assessment and comments.

**Section 10. Secondary outcomes**

S.Figure 1. Forest plot for change in psychotic symptoms in CHR

Results for PANSS symptom domains

S.Figure 2. Forest plot for change in PANSS Positive in FEP

S.Figure 3. Forest plot for change in PANSS Negative in FEP

S.Figure 4. Forest plot for change in PANSS General in FEP

S.Figure 5. Forest plot for change in PANSS Total in FEP

Classical vs non-classical anti-inflammatories

S.Figure 6. Forest plot for change in symptom scores in classical vs non-classical anti-inflammatories in FEP

Individual types of anti-inflammatories

S. Figure 7. Forest plot for change in symptom scores for each individual type of anti-inflammatory in FEP

Exclusion of Emsley 2014.

S.Figure 8. Forest plot for change in symptom scores for the exclusion of Emsley 2014.

**Section 11. Sensitivity analyses**

A. **Fixed-effect models for primary outcomes**

S.Figure 9. Forest plot for transition to psychosis in CHR

S.Figure 10. Forest plot for change in symptom scores in FEP

B. **Exploration of Heterogeneity with Meta-Regression**

Effect of ROB

Subgroup analysis showed that studies at **high risk of bias** reported a larger effect size (SMD = –0.61; 95% CI: –0.81 to –0.42), whereas studies rated as **some concerns** or **low risk** showed smaller and non-significant effects. The between-subgroup difference was not statistically significant under the random-effects model (Q = 1.29, p = 0.524), suggesting no strong evidence that risk of bias significantly moderated the effect.

S.Figure 11a. Forest plot for change in symptom scores in FEP grouped by Risk of Bias

S.Figure 11b. Forrest plot for exclusion of High ROB studies

For FEP, when we exclude studies with a high ROB, the effect size is smaller, and the results are no longer significant (SMD = -0.28, 95% CI: -0.89 to 0.34), raising concerns about low-quality studies overestimating effect sizes. For CHR, the non-significant results remain.

Sample size

A meta-regression was conducted to assess whether sample size was associated with variation in effect sizes across studies. The analysis included 12 studies and used a random-effects model (REML estimator).

The regression model showed that sample size was a significant moderator (QM = 13.94, p = 0.0002), explaining approximately 74% of the heterogeneity (R² = 74.24%). The regression coefficient was positive (β = 0.0067, 95% CI: 0.0032 to 0.0103, p=0.002), indicating that studies with larger sample sizes tended to report smaller (less negative) effect sizes, i.e., less benefit of the intervention.

Residual heterogeneity after accounting for sample size was reduced (I² = 40.15%), and the test for residual heterogeneity was non-significant (QE = 16.95, p = 0.076), suggesting the model accounted for most of the observed variability.

Year of publication

We conducted a meta-regression to evaluate whether the year of publication was associated with variation in effect sizes across the 12 included studies. A random-effects model with REML estimation was used.

The analysis found that year of publication was not a statistically significant moderator of effect size (QM = 1.37, p = 0.24). The regression coefficient was positive but non-significant (β = 0.0465, 95% CI: –0.0314 to 0.1245), indicating no clear trend in effect size over time.

The model explained only a small proportion of heterogeneity (R² = 7.66%), and substantial residual heterogeneity remained (I² = 73.51%), confirmed by a significant test for residual heterogeneity (QE = 46.16, p < .0001). This suggests that year of publication does not meaningfully explain the between-study variability.

Type of anti-inflammatory

To assess the impact of different types of anti-inflammatories on heterogeneity, we analysed both individual anti-inflammatory types and a broader classification of classical versus non-classical agents. The test for subgroup differences among individual types was significant (Q = 15.18, p = 0.0145), suggesting that the specific type of anti-inflammatory may contribute to the heterogeneity observed in the main analysis. However, several subgroups included only one study each, so these results should be interpreted with caution due to the potential influence of individual studies. In contrast, the meta-regression examining the broader classification (classical vs. non-classical) did not show a statistically significant difference between groups (Q = 0.49, p = 0.48).

Mean age of participants

Meta-regression did not identify age as a significant moderator of effect size (β = 0.017, p = 0.79), and did not explain any of the between-study heterogeneity (R² = 0%). Heterogeneity remained high (I² = 76.8%).

Duration of treatment

Meta-regression did not identify treatment duration as a significant moderator of effect size (β = 0.003, p = 0.65), and it did not explain any of the observed heterogeneity (R² = 0%). Heterogeneity remained high (I² = 76%).

C. **Leave-one-out analyses**

S.Table 2. Leave-one-out analysis for CHR

| Name | N | Heterogeneity I^2^ | Result |
| --- | --- | --- | --- |
| Total MA | 892 | 59% | 0.88 (0.42, 1.86) |
| Amminger 2010 | 81 | 0% | 1.20 (0.76, 1.90) |
| McGorry 2017 | 304 | 69% | 0.77 (0.26, 2.28) |
| Qurashi 2023 | 326 | 66% | 0.73 (0.26, 2.02) |
| Wasserthal 2024 | 46 | 74% | 0.95 (0.15, 5.91) |
| Winter-Van 2024 | 135 | 67% | 0.71 (0.15, 3.29) |

S.Table 3. Leave-one-out analysis for FEP

| Name | N | Heterogeneity I^2^ | Result |
| --- | --- | --- | --- |
| Total Meta-analysis | 886 | 81% | **-0.38 (-0.76, 0.00)** |
| Breier 2018 | 60 | 78% | -0.31 (-0.69, 0.08) |
| Deakin 2018 | 207 | 69% | **-0.46 (-0.83,-0.08)** |
| Emsley 2014 | 33 | 80% | **-0.46 (-0.81,-0.10)** |
| Husain 2024 | 68 | 81% | **-0.44 (-0.84,-0.04)** |
| Lui 2028 | 55 | 82% | -0.37 (-0.79, 0.05) |
| Muller 2010 | 50 | 81% | -0.34 (-0.76, 0.07) |
| Pawelczyk 2016 | 71 | 82% | -0.36 (-0.78, 0.06) |
| Pyatpykina 2020 | 18 | 80% | -0.31 (-0.67, 0.04) |
| Ritsner 2014 | 52 | 82% | -0.37 (-0.79, 0.05) |
| Robinson 2019 | 50 | 82% | -0.37 (-0.79, 0.05) |
| Sommer 2021 | 119 | 82% | **-0.42 (-0.84, 0.00)** |
| Zhang 2021 | 93 | 81% | -0.35 (-0.77, 0.07) |

D. **Effect of study size**

S.Figure 12. Funnel plot for CHR studies

S.Figure 13. Funnel plot for FEP studies

S.Figure 14. Trim and fit analysis funnel plot for FEP

E. Duration of illness less than 2 years

To facilitate comparison with previous meta-analyses that have defined first-episode psychosis (FEP) using a more restrictive time frame, we conducted a sensitivity analysis limited to studies in which participants had a duration of illness of two years or less. This cut-off has been used in several prior reviews, including Jeppesen et al. (2020), and is often considered a narrower definition of the early phase of psychosis.

Five studies met this criterion, either through inclusion criteria specifying ≤2 years since onset or by reporting a mean illness duration under two years. These studies were: Breier et al. (2018), Müller et al. (2010), Pawelczyk et al. (2016), Robinson et al. (2019), and Zhang et al. (2021).

The pooled effect size across these five studies was SMD = -0.71 (95% CI: -1.00 to -0.43), p = 0.002, with no observed heterogeneity (I² = 0%). This estimate is notably larger than that reported in the Jeppesen et al. (2020) review, which found an SMD of -0.26 (95% CI: -0.52 to -0.01) in a similarly defined early psychosis population.

While this result suggests a potentially stronger treatment effect in studies of individuals earlier in the course of illness, several important limitations must be considered. The analysis includes only five studies, limiting the robustness and generalisability of the findings. Moreover, the risk of bias was high in the majority of included studies: three were rated as high risk of bias using the ROB2 tool, one had some concerns, and only one study was rated as low risk.

Together, these limitations indicate that the larger observed effect size may reflect methodological weaknesses rather than a true underlying difference. As such, while this sensitivity analysis offers a useful point of comparison with prior literature, the results should be interpreted cautiously.

F. **Variation of means and coefficient results**

This forest plot presents the pooled meta-analysis of the log-transformed ratios of standard deviations (SDs) comparing treatment and control groups across studies. An SD ratio greater than 1 indicates greater variability in the treatment group, suggesting differences in how participants respond to the intervention. The random-effects model accounts for between-study heterogeneity.

S. Figure 15. Forest plot for the pooled Standard deviation variation for FEP studies

This forest plot shows the pooled meta-analysis of the log-transformed ratios of coefficients of variation (CVs) between treatment and control groups. The CV ratio standardizes variability relative to the mean, providing a measure of relative heterogeneity. A CV ratio above 1 suggests higher relative variability in the treatment group, which may reflect differential response to the intervention among participants.

S.Figure 16. Coefficient of Variance for FEP studies

**Section 14. GRADE assessment results.**

**Author(s):** ERP, MJT

**Question:** Anti-Inflammatories Compared to Placebo for First Episode Psychosis, Anti-Inflammatories Compared to Placebo for Clinical High Risk

| **Certainty assessment** | | | | | | | **№ of patients** | | **Effect** | | **Certainty** | **Importance** |
| --- | --- | --- | --- | --- | --- | --- | --- | --- | --- | --- | --- | --- |
| **№ of studies** | **Study design** | **Risk of bias** | **Inconsistency** | **Indirectness** | **Imprecision** | **Other considerations** | **Anti-Inflammatories** | **Placebo** | **Relative (95% CI)** | **Absolute (95% CI)** |  |  |
| **Change in Psychotic Symptom Measure (assessed with: PANSS/BPRS)** | | | | | | | | | | | | |
| 12 | randomised trials | serious^a^ | serious^b^ | serious^c^ | serious^d^ | publication bias strongly suspected^e^ | 458 | 418 | - | SMD **0.38 SD lower** (0.76 lower to 0.00 lower) | ⨁◯◯◯ Very low^a,b,c,d,e^ | CRITICAL  ^j^ |
| **Transition to Psychosis** | | | | | | | | | | | | |
| 5 | randomised trials | not serious^f^ | serious^g^ | serious^h^ | serious^i^ | none | 68/527 (12.9%) | 48/365 (13.2%) | **OR 0.88** (0.26 to 3.01) | **14 fewer per 1,000** (from 94 fewer to 182 more) | ⨁◯◯◯ Very low^f,g,h,i^ | CRITICAL  ^k^ |

**CI:** confidence interval; **OR:** odds ratio; **SMD:** standardised mean difference

#### Explanations

a. The majority of included studies were at low risk of bias, with some having some concerns. However, 4 out of 12 studies (34.2% of the total weight) were assessed as high risk of bias. Given that these studies contribute a substantial proportion of the overall evidence, their inclusion may introduce bias into the pooled estimate. Therefore, we downgraded the certainty of evidence by one level for serious risk of bias.

b. There is substantial heterogeneity (I² = 79%), primarily explained by differences in study size and year of publication, with newer studies having larger sample sizes and smaller effect sizes. Together, these factors explain nearly 80% of the heterogeneity. Additional contributors include risk of bias and variation in the type of anti-inflammatory used. While the studies show different results, the majority (10/12) favour the intervention, though only 4 studies report statistically significant findings (3 favouring intervention, 1 favoring control). Given that most heterogeneity is explained and the overall trend remains consistent, we downgrade by one level for serious inconsistency.

c. The population is appropriate, as all studies included individuals with first-episode psychosis (FEP). The comparator (placebo alongside standard antipsychotic treatment) is also relevant to clinical practice. However, indirectness is introduced by the variation in anti-inflammatory agents, which differ in mechanism of action, duration, and potential effects on psychotic symptoms. Despite this heterogeneity, the outcome measures are standardized, using only two widely accepted scales. Given these factors, we downgrade the certainty of evidence by one level for serious indirectness.

d. The pooled standardized mean difference (SMD) for change in psychotic symptoms was -0.47 (95% CI: -0.80 to -0.14), indicating a statistically significant effect in favour of anti-inflammatories. The total sample size across the 12 studies was 876 participants (458 in the intervention group and 418 in the control group), meeting the optimal information size (OIS) requirement. However, there are concerns regarding clinical significance. While the effect is statistically significant, the SMD of -0.47 represents a small effect size, which may have limited clinical relevance. Additionally, only 4 of the 12 studies reported statistically significant effects, increasing uncertainty in the precision of the pooled estimate. Given these considerations, we downgraded the certainty of evidence by one level for serious imprecision. However, we did not downgrade by two levels, as the confidence interval does not cross the null effect (0) and the total sample size is adequate.

e. Egger’s test for funnel plot asymmetry was statistically significant (t = -3.8882, p = 0.0030), indicating potential publication bias. Given this concern, we downgraded the certainty of evidence by one level for publication bias. No evidence of a dose-response relationship was found, meaning higher doses or longer durations of anti-inflammatory treatment did not show a consistent increase in effect. Additionally, no confounding was identified, reducing concerns about systematic biases affecting results. Finally, the pooled effect size (SMD = -0.47) is small, meaning the results do not meet the criteria for upgrading certainty based on a large effect. Considering these factors, we applied a one-level downgrade for serious publication bias but did not make further downgrades for other considerations.

f. The majority of included studies were at low risk of bias, with only two studies having some concerns related to missing data. One study (18% of the pooled weight) was assessed as high risk of bias due to missing data but was otherwise methodologically robust. Given that the overall risk of bias remains low and missing data does not appear to be a pervasive issue, we did not downgrade the certainty of evidence for this domain.

g. There was moderate heterogeneity (I² = 59%), and the direction of effects varied across studies. Of the five included studies, only one showed a statistically significant effect, while the others were non-significant. Additionally, the distribution of effects was inconsistent, with two studies favouring intervention and three favouring control. Given the lack of a clear trend and the variability in results, we downgraded the certainty of evidence by one level for serious inconsistency

h. While all included studies focused on UHR individuals, the intervention varied substantially, with different types of anti-inflammatories used (mostly PUFAs, but also NAC and an antibiotic). These treatments have different mechanisms of action, durations, and potential clinical effects, introducing concerns about whether the pooled estimate reflects a consistent treatment effect. Although the comparator (placebo) was appropriate and the outcome was measured using standardized methods, we downgraded the certainty of evidence by one level for serious indirectness due to intervention heterogeneity.

i. The pooled odds ratio was 0.88 (95% CI: 0.26 to 3.01), with a wide confidence interval crossing the line of no effect, suggesting uncertainty about the true effect. While 4 out of 5 studies did not show significant effects, the total sample size of 892 participants provides reasonable confidence in the estimate. Given the balance between imprecision and sample size, we downgraded the certainty of evidence by one level for serious imprecision.

References

1.	Julian PT Higgins, Sandra Eldridge, Tianjing Li. Chapter 23: Including variants on randomized trials. In: *Cochrane Handbook for Systematic Reviews of Interventions*. Version 6.5. ; 2024. Accessed May 29, 2025. https://training.cochrane.org/handbook/current/chapter-23

2.	Julian PT Higgins, Tianjing Li, Jonathan J Deeks. Chapter 6: Choosing effect measures and computing estimates of effect. In: *Cochrane Handbook for Systematic Reviews of Interventions*. Version 6.5. ; 2024. Accessed May 29, 2025. https://training.cochrane.org/handbook/current/chapter-06

3.	Amminger GP, Schäfer MR, Papageorgiou K, et al. Long-Chain ␻-3 Fatty Acids for Indicated Prevention of Psychotic Disorders. *ARCH GEN PSYCHIATRY*. 2010;67(2).

4.	Breier A, Liffick E, Hummer TA, et al. Effects of 12-month, double-blind N-acetyl cysteine on symptoms, cognition and brain morphology in early phase schizophrenia spectrum disorders. *Schizophr Res*. 2018;199:395-402. doi:10.1016/j.schres.2018.03.012

5.	Deakin B, Suckling J, Barnes TRE, et al. The benefit of minocycline on negative symptoms of schizophrenia in patients with recent-onset psychosis (BeneMin): a randomised, double-blind, placebo-controlled trial. *Lancet Psychiatry*. 2018;5(11):885-894. doi:10.1016/S2215-0366(18)30345-6

6.	Pawełczyk T, Grancow-Grabka M, Kotlicka-Antczak M, Trafalska E, Pawełczyk A. A randomized controlled study of the efficacy of six-month supplementation with concentrated fish oil rich in omega-3 polyunsaturated fatty acids in first episode schizophrenia. *J Psychiatr Res*. 2016;73:34-44. doi:10.1016/j.jpsychires.2015.11.013

7.	Pyatoykina AS, Zhilyaeva TV, Semennov IV, Mishanov GA, Blagonravova AS, Mazo GE. The double-blind randomized placebo-controlled trial of N-acetylcysteine use in schizophrenia: preliminary results. *Zhurnal Nevrol Psikhiatrii Im SS Korsakova*. 2020;120(9):66. doi:10.17116/jnevro202012009166

8.	Robinson DG, Gallego JA, John M, et al. A potential role for adjunctive omega-3 polyunsaturated fatty acids for depression and anxiety symptoms in recent onset psychosis: Results from a 16 week randomized placebo-controlled trial for participants concurrently treated with risperidone. *Schizophr Res*. 2019;204:295-303. doi:10.1016/j.schres.2018.09.006

9.	Husain MO, Chaudhry IB, Khoso AB, et al. Add-on Sodium Benzoate and N-Acetylcysteine in Patients With Early Schizophrenia Spectrum Disorder: a Multicenter, Double-Blind, Randomized Placebo-Controlled Feasibility Trial. *Schizophr Bull Open*. 2024;5(1):sgae004. doi:10.1093/schizbullopen/sgae004

10.	Müller N, Krause D, Dehning S, et al. Celecoxib treatment in an early stage of schizophrenia: Results of a randomized, double-blind, placebo-controlled trial of celecoxib augmentation of amisulpride treatment. *Schizophr Res*. 2010;121(1-3):118-124. doi:10.1016/j.schres.2010.04.015

11.	Winter-van Rossum I, Slot MIE, van Hell HH, et al. Effectiveness of Omega-3 Fatty Acids Versus Placebo in Subjects at Ultra-High Risk for Psychosis: the PURPOSE Randomized Clinical Trial. *Schizophr Bull*. Published online 2024. doi:10.1093/schbul/sbae186

12.	Zhang Y, Shi H, Yang G, et al. Associations between expression of indoleamine 2, 3-dioxygenase enzyme and inflammatory cytokines in patients with first-episode drug-naive Schizophrenia. *Transl Psychiatry*. 2021;11(1):595. doi:10.1038/s41398-021-01688-x

13.	Ritsner MS, Bawakny H, Kreinin A. Pregnenolone treatment reduces severity of negative symptoms in recent‐onset schizophrenia: An 8‐week, double‐blind, randomized add‐on two‐center trial. *Psychiatry Clin Neurosci*. 2014;68(6):432-440. doi:10.1111/pcn.12150

14.	McGorry PD, Nelson B, Markulev C, et al. Effect of ω-3 Polyunsaturated Fatty Acids in Young People at Ultrahigh Risk for Psychotic Disorders: The NEURAPRO Randomized Clinical Trial. *JAMA Psychiatry*. 2017;74(1):19. doi:10.1001/jamapsychiatry.2016.2902

15.	Schünemann HJ, Brennan S, Akl EA, et al. The development methods of official GRADE articles and requirements for claiming the use of GRADE – A statement by the GRADE guidance group. *J Clin Epidemiol*. 2023;159:79-84. doi:10.1016/j.jclinepi.2023.05.010

16.	Conus P, Seidman LJ, Fournier M, et al. N-acetylcysteine in a Double-Blind Randomized Placebo-Controlled Trial: Toward Biomarker-Guided Treatment in Early Psychosis. *Schizophr Bull*. 2018;44(2):317-327. doi:10.1093/schbul/sbx093

17.	Jeppesen R, Christensen RHB, Pedersen EMJ, et al. Efficacy and safety of anti-inflammatory agents in treatment of psychotic disorders – A comprehensive systematic review and meta-analysis. *Brain Behav Immun*. 2020;90:364-380. doi:10.1016/j.bbi.2020.08.028
